# Dental pedagogy in the ‘new normal’ COVID-19 era: a transition template of teaching protocols

**DOI:** 10.1186/s12909-022-03864-z

**Published:** 2022-11-16

**Authors:** Nadia Khalifa, Lakshman Samaranayake, Kausar Sadia Fakhruddin

**Affiliations:** 1 Department of Preventive and Restorative Dentistry, College of Dental Medicine, University of Sharjah, Sharjah, UAE; 2 Faculty of Dentistry, The University of Hong Kong, 34 Hospital Road, Pok Fu Lam, Hong Kong China

**Keywords:** Dental pedagogy, COVID-19, Clinical teaching, Modified Operating Procedure

## Abstract

**Aims:**

Delivery of clinical dental education, as opposed to clinical medicine, is particularly challenging due to the obligatory aerosol-generating procedures (AGPs) used in dentistry, which are known to facilitate the spread of severe acute respiratory syndrome coronavirus 2 (SARS-CoV-2). Hence, using AGPs and working in close proximity to patients for extended periods in dental hospital/university settings with multiple teaching clinics have been a formidable prospect for all stake holders. Therefore, several professional and governmental organizations have promulgated variations of infection control guidelines for general practice dentistry in the pandemic era to mitigate SARS-CoV-2 transmission.

**Materials and methods:**

In the absence of unified guidelines for modified infection control/clinical procedures for dental education. We implemented a clinical protocol template and modified operating procedures (MOP) for teaching clinical dentistry to fit the infection control requirements during the pandemic/post-pandemic period at the Sharjah University, College of Dentistry, UAE. MOPs ranged from various engineering control measures (e.g., negative-pressure ventilation systems in operatories) to administrative control measures featuring post-procedure fallow periods of treatment-abeyance between patient sessions.

**Results:**

The new MOPs for clinical dentistry in the COVID-19 pandemic era, trialled in a UAE dental teaching hospital, have successfully eliminated infection transmission amongst the students, clinicians, ancillary staff, or attending patients, thus far.

**Conclusions:**

The proposed MOPs that complement the standard operating protocols in clinical dentistry were an attempt to mitigate nosocomial infection transmission and protect four different groups of stakeholders, i) the patients, ii) the dental students, iii) the clinical academics, and iv) the para-dental personnel/assistants. Due to the endemicity of the COVID-19 in many regions of the World, the suggested MOPs need periodic review and revision, to fit the emerging data on the disease. Finally, as there are no studies to date comparing the relative efficacy of the MOPs in various dental academic institutions, there is an urgent need for future workers to address this issue.

## Background

World Health Organization (WHO), in January 2020, announced a new human pneumonia-like outbreak in Wuhan, China, termed the coronavirus disease 2019 (COVID-19), caused by the severe acute respiratory syndrome coronavirus 2 (SARS-CoV-2) infection, a global pandemic of unprecedented proportions. The ravaging impact of SARS-CoV-2 was profound, with the global population under ‘lockdown’ conditions in several countries [1]. In addition, there is now clear evidence to demonstrate SARS-CoV-2 transmission from person to person through many vectors, such as aerosols, respiratory droplets, and contact spread [2, 3] into unsuspecting victims via entry portals such as the nose, mouth, and eyes [4].

The major impediment for reining in the COVID-19 pandemic was the high prevalence of up to 40% asymptomatic disease carriers in the community. Radical containment measures to limit disease transmission have included social distancing, thorough hand washing, quarantine, and the use of personal protective equipment such as masks [5]. In addition, a raft of vaccines for COVID-19, and rapid diagnostics, such as RAT and PCR tests [6], are other preventive measures now in place [5, 7].

Compared to the general population at large, healthcare workers (HCWs), including dental professionals, run a greater risk of contracting respiratory pathogens by the nature of their profession. This was evident in the SARS epidemic of 2013, which led to numerous deaths of HCWs [8] and in the current COVID-19 pandemic, where HCWs were disproportionately affected [9]. Thankfully, there are no reports, to date, of mortality amongst dental healthcare workers (DHCWs) with workplace COVID-19 transmission. However, they were the professional group that was thought to have the highest likelihood of acquiring the infection in comparison to other clinical professions [10], according to the World Health Organization (WHO) [11].

It is now clear that COVID-19 will be entrenched in the global community for the foreseeable future, as an endemic disease. Given this scenario, dental educators need to rethink and reinvent effective and safe delivery modes of clinical dental education. As opposed to clinical medicine, the latter is particularly challenging in this context due to the inevitable and obligatory aerosol-generating procedures (AGPs) used for dental patient management while working for prolonged periods in close proximity to patients who may be unknowingly harbouring the SARS-CoV-2. For instance, it is well known that in dentistry, the routine use of high-speed handpieces with water jets, air/water syringes, ultrasonic scaling, and air polishing causes such aerosolization of microbes into the ambient clinic air [12].

## Dentistry in a teaching clinic in UAE

After the pronouncement by the World Health Organization that SARS-CoV-2 infection is a pandemic, on 11th of March, 2020, the Ministry of Health and Prevention, in liaison with the Ministry of Education of the United Arab Emirates (UAE), advised the cessation of clinical dentistry, apart from urgent procedures, in all primary care dental practices in the country. The university dental colleges followed these instructions in UAE, and all non-clinical teaching was moved to virtual platforms. Furthermore, university examinations were rescheduled and remodelled, and criteria for the successful graduation of students were reviewed and modified considering the prevailing conditions.

This situation persisted until mid-July 2020, after which UAE authorities decided to resumption dental care delivery in accordance with stringent guidelines provided by international and national health regulatory bodies [13]. The New operational model described here, which supplemented the Standard Operating Procedures in clinical dentistry, was an attempt to mitigate infection transmission to four different groups of stakeholders, namely i) the patient, ii) the dental student, iii) the clinical academic, and iv) the para-dental personnel/assistants.

In the absence of standard universally promulgated guidelines on obviating COVID-19 nosocomial transmission in the dental educational environment, we provide salient features below of a new operational model for a dental hospital teaching facility in UAE. These guidelines could be used to offer protection to all stakeholders while simultaneously delivering safe and efficacious clinical care for the patient and uninterrupted education for aspiring dentists.

## The profile of the dental teaching hospital

University Dental Hospital Sharjah-UAE (UDHS-UAE) is a primary dental healthcare facility equipped with 124 dental units. It is home to more than 125 academic and non-academic staff and approximately 186 undergraduate clinical students. During the pre-COVID era, the hospital provided comprehensive dental care to some 150 patients daily, during two clinical sessions of 3 hours each. Using a curriculum based on the comprehensive dental care delivery teaching model, the undergraduate trainees and residents were mentored to care for all walk-in patients irrespective of their socio-economic status.

## Mitigation of nosocomial infection during COVID-19

In considering infection control in an institutionalized setting or otherwise, the analytical and subsequent decision-making processes should generally be based on the following three main principles.

3.1. *Administrative controls* offer work policies and procedures to reduce the exposure of all stakeholders to the identified hazard and change the way people work.

3.2. *Engineering controls* entail changes in the physical features of the workplace, such as hospital design and configuration, to mitigate pathogen exposure at the source and improve compliance.

3.3. *Personal protective equipment (PPE)* utilization was considered with engineering solutions and administrative controls (Note: PPE is not described here as it is beyond the remit of this article).

**Fig. 1:**
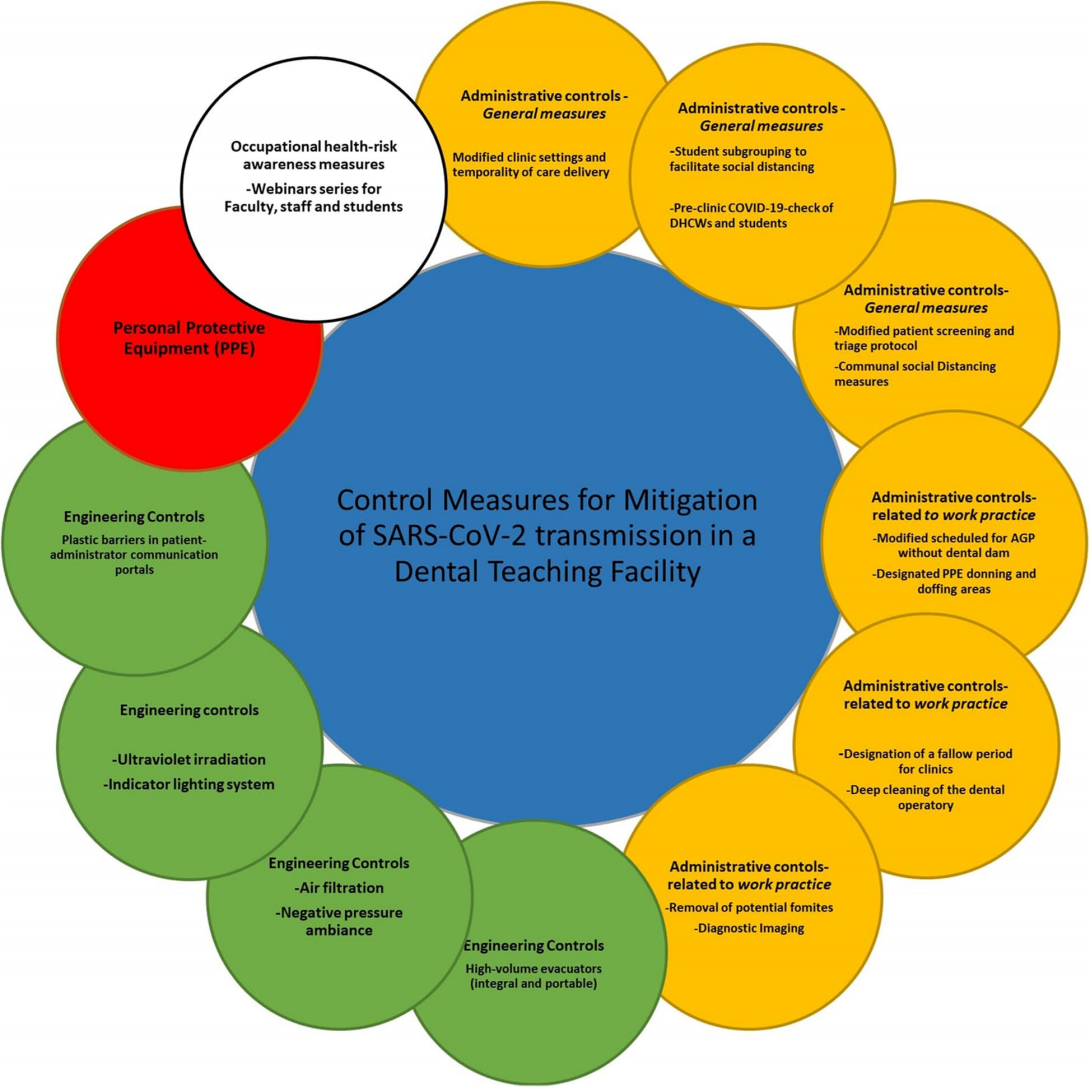
Nosocomial infection risk management framework recommended in a Dental Teaching Facility during the COVID-19 pandemic

The following describes in detail how the MOPSs entailing engineering and administrative control measures were planned and implemented at the University Dental Hospital Sharjah, UAE (UDHS-UAE) (Fig. 1).

### Administrative controls

The administrative controls were sub-categorized as a) general measures and b) those appertaining to work practices. These are outlined below.

#### Administrative controls - general measures

##### Modified clinic settings (physical changes at the workplace) and temporality of care delivery

**Fig. 2:**
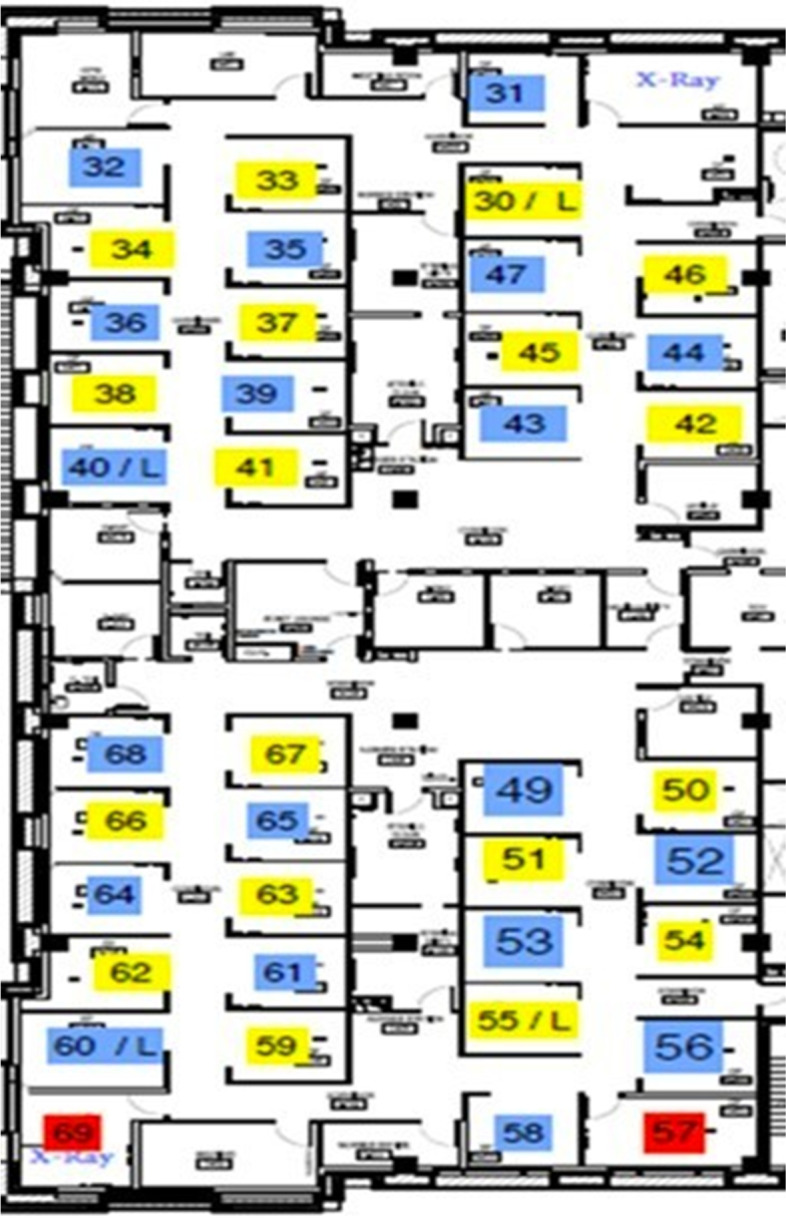
The configuration of clinic chairs during the pandemic

The UDHS-UAE dental clinic’s architectural profile is the standard model, featuring multiple, partially isolated cubicles set in clusters and rows, with separate clinics devoted to specialized care. The new guidelines for infection mitigation entailed alternate colour-coding of each cubicle, either as blue or yellow, with 18-blue-coded functioning units alternating with an equivalent number of yellow-coded units (Fig. 2). To maintain the social distancing ethos, this initiative ensured that only a single-colour coded blue or yellow clinic was operational at a given time, with no adjacent cubicles used for simultaneous patient care.

As a consequence of this administrative control, the clinic’s functional capacity was reduced by 50%, leading to knock-on issues that entailed longer teaching hours extending well into the late evening with modified, sub-divided, multiple clinical sessions for each student group. In practical terms, the blue/yellow coded cubicle arrangement permitted a more than 4-m space between two functioning units, thus maintaining a safe, social distancing system to obviate infection transmission. This arrangement also means that the alternate cubicles can be deep cleansed with the increased fallow period in between patient sessions (see below).

##### Student subgrouping to facilitate social distancing

**Fig. 3:**
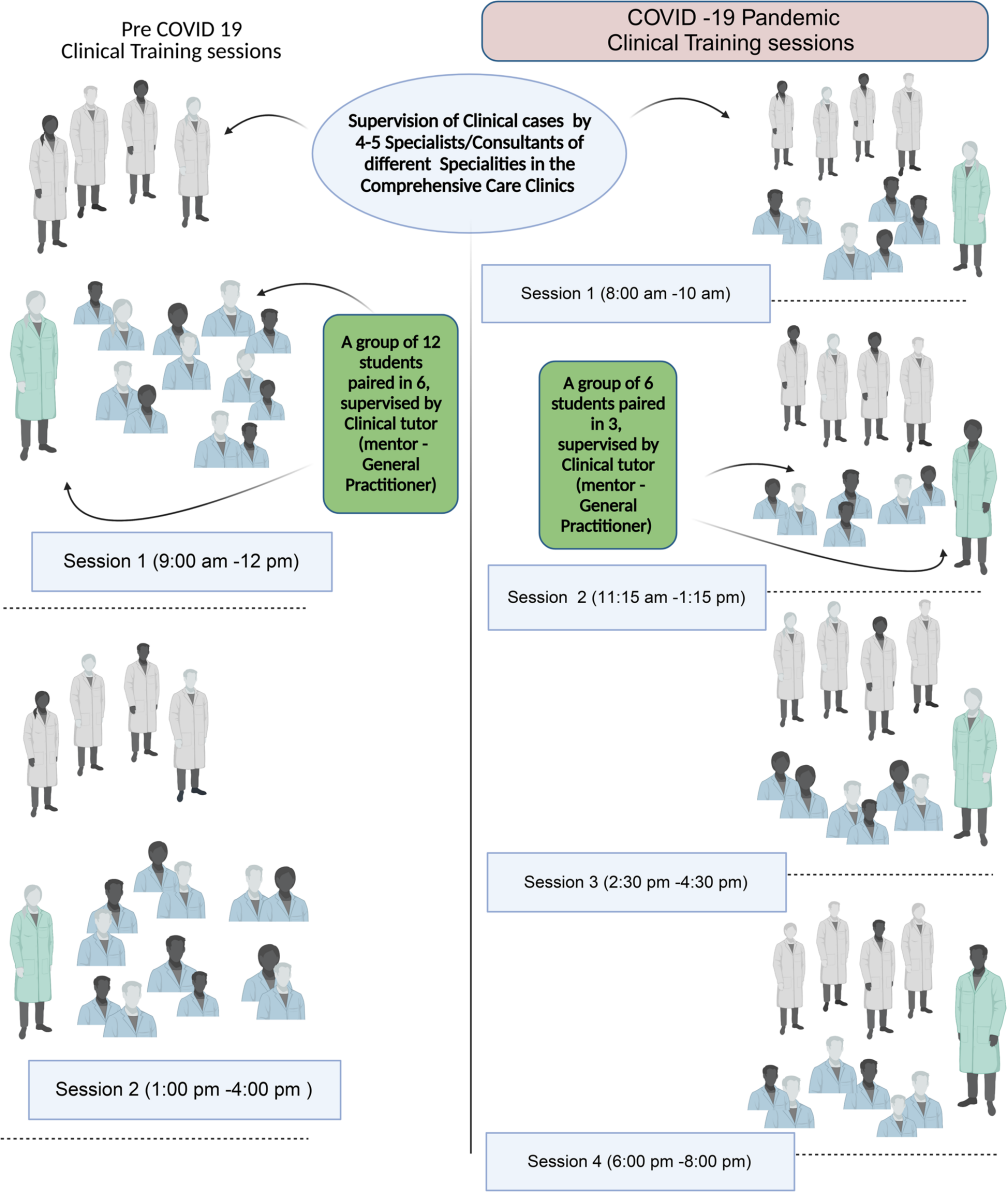
Clinical training management, session distribution, and workflow before and during the COVID-19 pandemic

During the pre-COVID era, the students of BDS years four and five were paired (the buddy system) to deliver clinical care guided by four-handed dentistry philosophy from 9 am to 4 pm. However, these new teaching arrangements of split teaching sessions and stretched teaching periods and clinical sessions well into the evening hours (8 am to 8 pm) necessitated an increased workforce, such as general practitioners and additional specialists from different dental specialties (Fig. 3).

##### Pre-clinic COVID-19-check of DHCWs and students

In the UAE, the government has mandated a contact tracing application, ALHOSN, which utilizes BlueTooth technology to swiftly monitor and alert individuals of their COVID-19 health status [14]. This was accomplished through a QR-based colour-coded system that is verifiable and can be scanned and refreshed every 2 minutes.

A unique QR code may comprise one of four colours (grey, green, red, or amber). An untested individual displays a ‘grey’ QR code. In contrast, a negatively tested or healthy individual with a ‘green’ code grants the individual access to all public spaces. On the contrary, a ‘red’ QR code signifies a ‘COVID-positive’ individual. An ‘amber’ QR code implies a test or re-test for possible COVID-19 exposure. Accordingly, only the ‘green’ QR-coded faculty members, administrative and clinical staff, students, and resident dentists are eligible to enter the hospital premises after they are temperature scanned at the hospital’s designated, dedicated entry point.

Besides temperature checks, hand sanitization at the designated entrance for DHCWs, teachers, and students were required. In addition, all DHCWs were mandated to wear full enhanced personal protective equipment (PPE), including an N95 respirator, protective eyewear, face shield, disposable gowns, and protective layering on footwear in designated PPE donning areas (see below).

##### Modified patient screening and triage protocol

Patient entrance to the hospital was organized via a designated entry point after passing through an installed ‘sanitizing tunnel,’ leading to a ‘fever check’ booth. Following hand sanitization and fever check, the designated resident dentist in full enhanced PPE recorded COVID-19-related symptoms (if any) from the patients using a ‘patient triage questionnaire.’ Only patients that showed apparent COVID-negative symptoms were allowed in institution.

In the event of a COVID-19 suspect or positive patient being identified, emergency care was provided. The designated nurse escorted these patients to the negative pressure ‘isolation clinic’ dedicated for emergency dental procedures. Furthermore, a suspected COVID-19 case with non-emergency dental needs was referred to the adjoining, designated Emergency Care Department of the Medical Facility of the Sharjah University Hospital, which has special facilities for suspected COVID-19 patients.

##### Communal social distancing measures

The communal seating in the waiting area was arranged to permit a social distance of 6-ft between two seated patients [11]. Additionally, only a single essential visitor accompanying the patient was allowed within the hospital premises, either in the communal waiting area or within the clinical bay, for necessary assistance during the patient treatment session. Likewise, faculty, staff, and students were only permitted into hospital premises only when scheduled. Finally, upon patient discharge from the clinic, the patient and the attendee, if any, were escorted out of the hospital premises immediately afterwards by an assigned staff member.

#### Administrative controls related to work practices

##### Modified scheduled for AGP without dental dam

Where possible, students were encouraged to avoid AGPs, using hand excavators for carious dentine excavation and hand instruments for scaling and root planing. The use of a rubber dam was essential. If dental procedures such as ultrasonic scaling, subgingival restorations, or surgical procedures that generate copious bioaerosols cannot be avoided and rubber dam utilization was not possible [15–17], then these specific treatments were scheduled towards the end of the session to allow for a fallow period of treatment abeyance.

##### Designated PPE donning and doffing areas

Wearing (donning) personal protective equipment (PPE) before a clinical session and removing (doffing) them afterwards need to be done with extreme care, as the newly donned PPE should not be contaminated. In contrast, in the post-clinic session, contaminated PPE should be discarded not to contaminate any other source with which they come into contact. Hence separate donning areas (clean areas) and doffing areas (dirty areas) should be designated in any dental clinic. This situation was further complicated when numerous students and teaching and para-dental support staff work in a multi-unit teaching clinic. Consequently, distinct designated areas for the ‘donning’ and ‘doffing’ of personal protective equipment (PPE) were created in each teaching clinic. It was critical to have either adequate natural or negative pressure ventilation in such areas that should be large enough to accommodate social distancing.

##### Designation of fallow periods for clinics

A period of treatment abeyance between two different patient sessions in a clinical unit is termed the fallow period. The fallow period needs to be determined and guided by the clinical procedure undertaken, as well as the use of AGP and ambient ventilation. UK dental authorities had provided extensive guidance on determining and calculating the fallow period [18]. For instance, a clinical procedure using a rubber dam, AGP, and HEPA filtration evacuation should have a 45 min fallow period [19]. The designated fallow periods in our teaching clinics were 3 hours because of the alternate use of working cubicles.

##### Deep cleaning of the dental operatory

Following the fallow period, the assigned nursing team dons appropriate PPE and disinfects operatories with particular attention to high-contact surfaces, utilizing appropriate hospital-grade disinfectants (Viruton® Forte, MEDISEPT) prior to the arrival of the next patient.

##### Removal of potential fomites

All unnecessary shelving, furniture, brochures, pamphlets, and magazines that may surreptitiously act as fomites for vector transmission of infection were removed from the clinical areas and the hospital lobby. These inanimate objects or fomites frequently handled by clinic attendees and staff cannot be easily sanitized and are known to be potential vectors of viral transmission [11].

##### Diagnostic imaging

COVID-era recommendations from local and international regulatory bodies are to defer or avoid intraoral radiographs as much as possible during the pandemic. Such procedures frequently evoke coughing or gagging reflex in the patient, thereby generating microbe-laden aerosols [20, 21]. Hence, intraoral procedures in the imaging units were replaced by extraoral bitewings, extraoral lateral oblique view, and sectional or full-width dental panoramic (OPG) radiography as alternative imaging techniques [21] whenever possible.

### Engineering controls

Many interventional procedures are known to aerosolize respiratory secretions in healthcare settings [12, 22]. In dentistry, viral particles may be aerosolized by the high-speed handpiece and the accompanying air jet, ultrasonic scaling, air polishing, and air/water syringes. To reduce bioaerosol generation, especially in settings where multiple patient treatment sessions are simultaneously conducted [12], as in dental teaching hospitals, the following control measures are recommended i) extra-oral high-volume evacuation, ii) negative pressure ventilation, iii) air filtration, iv) ultraviolet irradiation of the operatories v) plastic barriers in patient-administrator communication portals vi) red-green lighting system in each cubicle to indicate the use of AGPs [23, 24].

#### High-volume evacuators (integral and portable)

It is possible to mitigate the burden of bio-aerosolization in the operatories by installing High-volume evacuators HVE for the operatories during AGP, along with retrofitted filters, which are integral components of the hospital’s air conditioning system [25].

#### Air filtration

High-efficiency particulate air (HEPA) filters incorporated into existing centralized ventilation systems (HVAC) can decontaminate the clinics’ ambient exhaust air [11]. HEPA filters with MERV-13 level (Minimum Efficiency Reporting Values) mechanically trap particles on impact and capture 99.97% of 0.3-μm contaminants that pass through the filters [26].

#### Negative pressure ambience

Creating negative air pressure in clinics helps keep airborne viruses and other microbes from entering the surrounding rooms and hallways, thus decreasing the potential risk of COVID-19 spread from clinics [27].

#### Ultraviolet irradiation

Ultraviolet (UV) germicidal irradiation utilizes short wavelength UV light to inactivate microbes, including viruses, essentially disrupting their nuclear DNA/RNA. They are generally harmless for humans unless on exposure for prolonged periods [28].

#### Plastic barriers in (patient-administrator) communication portals

Further engineering measures to restrict close contact between administrative staff and patients in the reception areas included installing a transparent plastic/glass barrier between the receptionist and the patient with a communication portal.

#### Indicator lighting system

Another ancillary engineering control measure was to install an indicator lighting system in each cubicle where a green light implied non-AGPs, and a red light indicated AGPs being in current use.

#### Occupational health-risk awareness measures

Last but not least, behavioural changes of the dental health care workers (DHCWs), including students, were a prerequisite for the proper execution of infection control measures [29]. Awareness of updated infection control methods related to occupational health risks through the ‘webinar’ series was organized for dental staff and students on a regular basis. Additionally, infographics demonstrated the appropriate steps for donning and doffing PPE displayed in the assigned areas. Furthermore, infographics and posters on the pandemic’s current status were regularly posted in all relevant areas of the hospital and displayed on the television screens in the patient waiting areas.

##### Logistic management during the COVID-19 pandemic

While many dentistry colleges across the globe chose to close the clinics and delay student graduations due to the pandemic, ours did not. Alternatively, efforts were invested in continuing the clinical and pre-clinical training sessions for our students in accordance with the constraints. Abiding by social distancing practices, students were distributed into subgroups, with labs and clinical sessions spread out, which stretched the teaching hours considerably. Supervision was further individualized due to student subgrouping. Therefore, hiring additional part-time staff was prioritized, so that the overall competency requirements were not compromised.

**Fig. 4:**
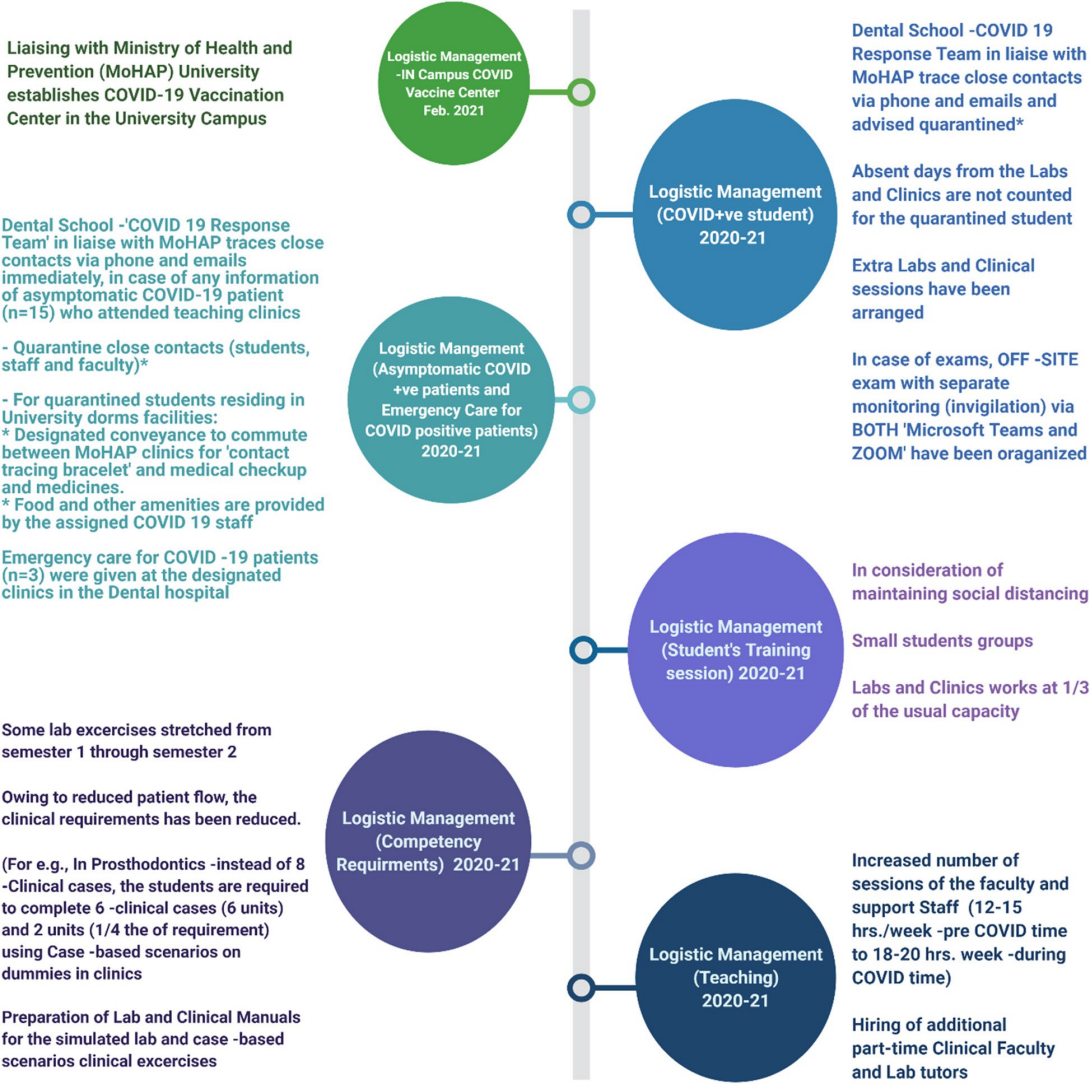
Employed changes in the logistic management of the Dental school and Dental Teaching Hospital during the COVID-19 pandemic

In consideration of the reduced patient flow consequent to the pandemic, clinical requirements were adjusted with the inclusion of case-based scenarios performed using ‘dummies’ in clinics. As a result, additional lab and clinical manuals were prepared by faculty, seen illustrated in Fig. 4.

**Fig. 5:**
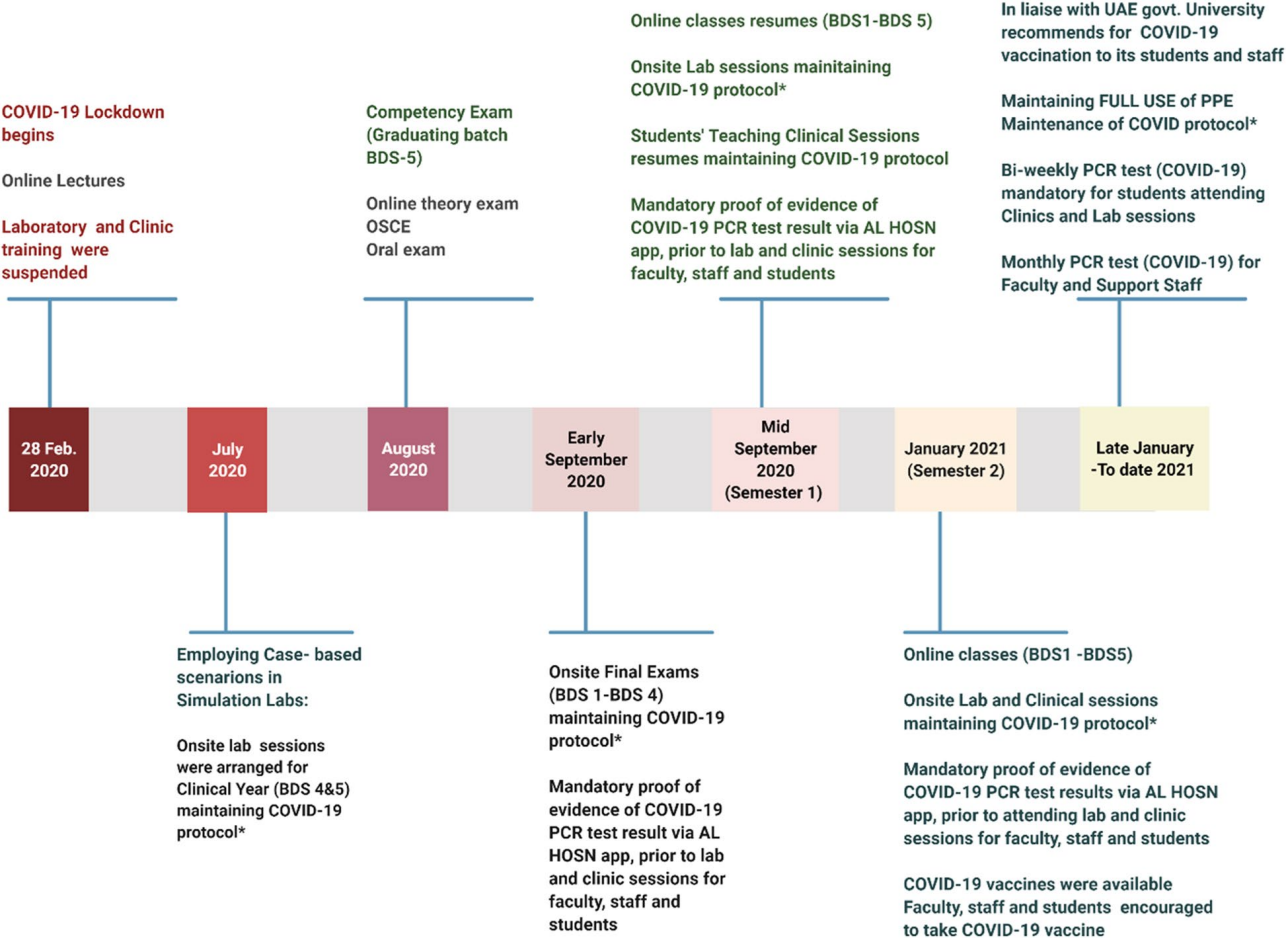
Different stages of COVID-19 pandemic – adapted educational measurements during the highly changing situations

Furthermore, as illustrated in the timeline in Fig. 5, exams and classes were resumed on time without delay. Even upon the arrival of the vaccines, which were available within the university premises, daily PCR tests remained mandatory for all staff and students alike. This has remained especially so in consideration of variant risk (Fig.5).

Students who contracted COVID were not marked as absent and were provided extra resources to complete their requirements missed during the quarantine period. In addition, the off-site examinations were arranged for them with additional invigilation staff devoted for the task.

Such extension of clinical periods positively impacted the continuity of patient care and the management of dental emergencies. It is noteworthy that our logistic policies and procedures were guided by the regulations promulgated by a number of other global health regulatory bodies and institutions [10, 30–32]. Clearly, therefore, many variations of the MOP described above have been implemented in various jurisdictions. However, it is difficult to ascertain their relative success or failure, first because it is difficult to make direct comparisons due to the variations in COVID-19 prevalence in different geographic regions. Second, as each jurisdiction may have different yardsticks for evaluating the success of disease control. To the best of our knowledge, there are no studies to date comparing the relative efficacy of various MOPs that are prevalent in various dental academic institutions, and there is an urgent need for future workers to address this issue.

## Conclusions

COVID-19 pandemic, has now taken root as an endemic infection with much less severity in many regions of the world due to the efficacious vaccination programs and a plethora of other social restrictions. This ‘new normal’ implies that the community as a whole need to live and conduct their daily business in the presence of a relentlessly mutating virus with variants emerging periodically. Therefore, a correct balance needs to be struck to offer novice students the necessary training while not compromising on the infection control measures needed for their safety as well as the teaching staff, and the patients. Hence the structure of the modified operative procedures (MOP) implemented in the University Dental Teaching Hospital Sharjah, described in detail here, should serve all stakeholders in the ‘dental’ community as a prudent template to mitigate infection spread now and into the future. The foregoing MOP describes in detail, how the various challenges were addressed, particularly the administrative and engineering controls required to prevent nosocomial spread of SARS-CoV-2 in a medium-sized dental teaching hospital. Although, the financial resources needed for engineering controls are substantial and burdensome, the administrative controls did not require similar outlays.

## Data Availability

Not Applicable, as this is a report on the management strategies adopted during the current COVID-19 pandemic in the Dental Educational Establishment. However, if required, the corresponding author can provide data included in the present study on reasonable request.

## Abbreviations

MOP: Modified Operative Procedure
UVGI: Ultraviolet Germicidal Irradiation
HEPA: High-Efficiency Particulate Air
AGP: Aerosol-Generating Procedure
ALHOSN: UAE COVID-tracking App
DHCWs: Dental Healthcare workers
UDHS: University Dental Hospital Sharjah
